# Prevalence and trend of TB/HIV co-infection in Suhum Municipality, Ghana

**DOI:** 10.1371/journal.pgph.0000378

**Published:** 2022-07-01

**Authors:** Haruna M. Salisu, Inumanye N. Ojule, Foluke O. Adeniji, George K. Kwakye

**Affiliations:** 1 School of Public Health, University of Port Harcourt, Port Harcourt, Nigeria; 2 Projects Department, Ghana Tourism Development Company, Accra, Ghana; 3 Department of Community Medicine, University of Port Harcourt Teaching Hospital, Port Harcourt, Nigeria; University of Embu, KENYA

## Abstract

Tuberculosis (TB) and Human Immunodeficiency Virus (HIV) infections have been identified to form a deadly synergy that is posing serious threats to human health and economic development particularly in Sub-Saharan Africa (SSA). Six years into the end TB strategy, it is imperative to assess HIV detection rate among TB patients in order to determine the prevalence as well as establish the temporal trend of the co-morbidity in the Eastern region of Ghana where the magnitude of HIV and TB/HIV co-morbidity have consistently been noted to be high. The study reviewed records of 840 TB patients retrospectively from January 1, 2009 to December 31, 2018 in Suhum Municipal. Socio-demographic characteristics and clinical data of study participants were extracted from the Municipal TB registers using an excel spread sheet. Data were exported into STATA version 16.0 for analysis with statistical significance set at p-value ≤0.05. Of the 840 TB patients, 793 (94.4%) were screened for HIV, with 18.6% (95% CI: 0.16–0.21) yielding positive results. A sharp increase in the trend of the co-infection was observed from 6 (14.6%) in 2009 to 21 (36.8%) in 2010. The highest (40.4%) co-infection prevalence was recorded in 2011. The study recorded an overall decreasing trend of the co-infection. Case detection rate for HIV among persons living with TB was high. TB/HIV co-infection rate in Suhum Municipal is high and occurs more often among females and persons aged 30 years to 49 years. A fairly stable prevalence trend of TB/HIV co-infection rate was also identified. In conclusion, ongoing integrated TB/HIV activities are showing good results and therefore need to be sustained.

## Introduction

Tuberculosis (TB) and Human Immunodeficiency Virus (HIV) infections remain health threats affecting all age groups and both sexes World-wide. Reports from the 2019 joint United Nations Programme on HIV/AIDS [1] indicate that TB continues to be a leading cause of premature morbidity and mortality from a single transmissible disease agent globally and claimed an estimated 4,400 lives a day out of 10 million people that fell ill with TB in the same year [2].

On the other hand, Human immunodeficiency virus disease another global epidemic, continues to be a major public health problem having claimed 36.3million (27.2–47.8million) lives to date [1]. At the end of 2020, there were an estimated 37.7 million (30.2–45.1million) people living with HIV, over two thirds of these are in the WHO African Region [2].

The negative synergistic relationship between TB and HIV infections has been aptly described by epidemiologists as a deadly syndemic. This relationship has tremendously increased healthcare challenges with huge impact on lives and development of people in Sub-Saharan Africa (SSA) [3]. What is often observed by clinicians all over the world is that persons with HIV have weakened immune system resulting in increased susceptibility to TB and other opportunistic infections. Conversely, for persons with TB also a major cause of suboptimal immunity, infection with HIV increases the risk of progression to active TB disease as well as re-activation of latent or dormant TB [4].

The burden due to co-infection varies markedly among countries and regions nonetheless, the heaviest burden of the co-morbidity has long been reported from SSA where the co-infection continues to cause devastating effects among human population [5].

Global efforts and partnerships have achieved good results in reducing the impact of both diseases. In Ghana also, what has been noted by statisticians is a downward trend of HIV prevalence such that there was a decrease from 2.4% in 2016 and 2.1% in 2017 to 2.0% in 2019 [6]. Deductively, a decrease in HIV prevalence is a pointer to a similar trend in TB prevalence. Within Ghana regional variations is however common. A 2020 hospital-based study from Greater Accra reported TB/HIV prevalence of 22.9% [7]. More so, population studies from the Volta region reported TB/HIV prevalence of 18.2% and 19.1% respectively in 2017 and 2019 [5, 8].

Even though the National TB control programme (NTP) and the Ghana AIDS commission are collaboratively implementing policies and monitoring activities to control the burden of the co-morbidity across the various regions in the country. It is expedient therefore, six years into the end TB strategy, which aims to reduce mortality and incidence rates due to TB by 95% and 90% respectively by 2035 compared to the 2015 levels, to monitor progress and report on the case detection rate and trend of TB/HIV co-infection in the Suhum Municipality in the Eastern region of Ghana where HIV prevalence is noted to be high.

## Materials and methods

### Study site

**Fig 1:**
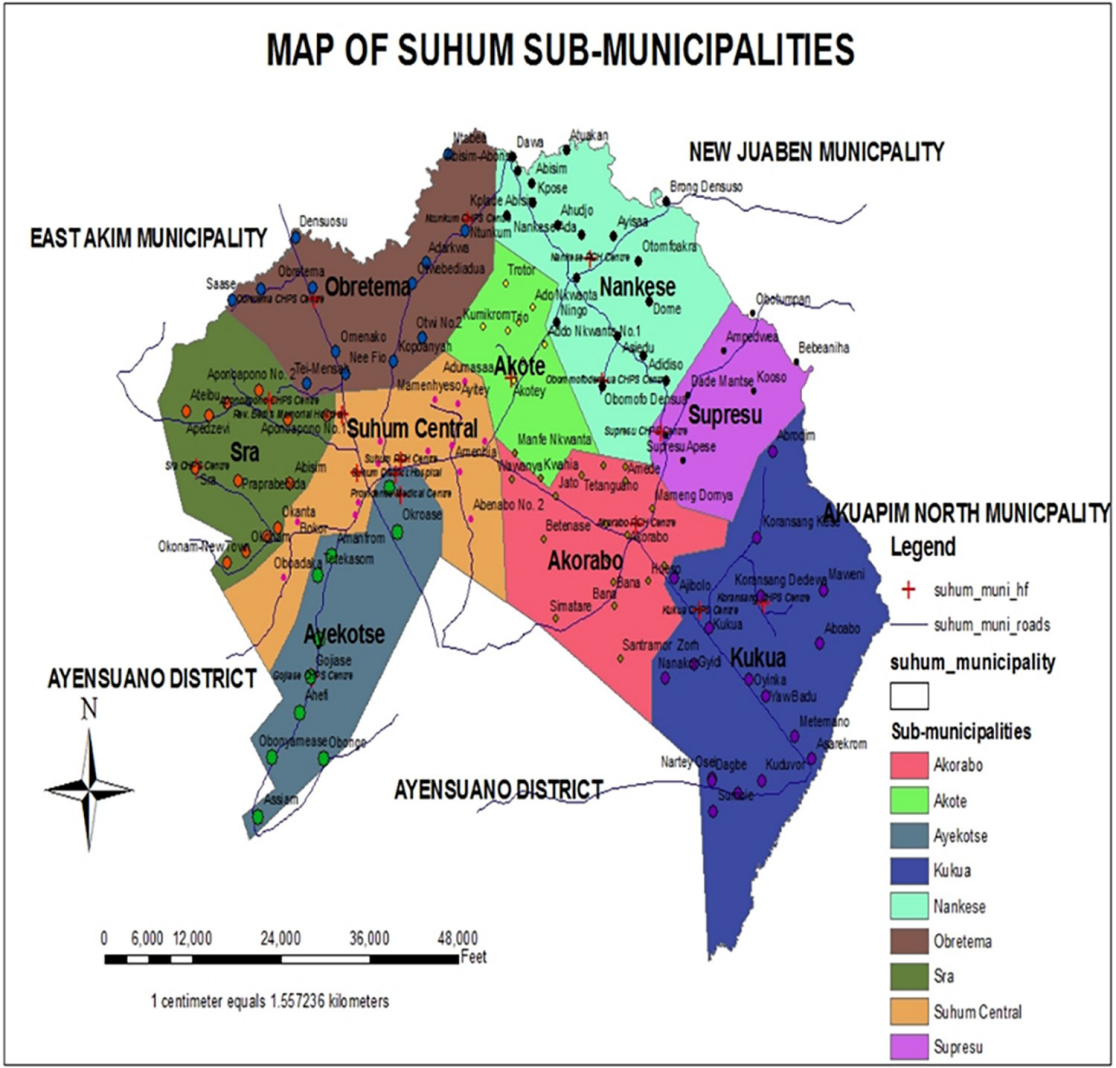
Map of Suhum Municipal. Source: Ghana Health Service, Suhum Municipal Profile (unpublished).

As shown in Fig 1, the study was carried out in Suhum Municipality, one of the 170 Metropolitan and Municipal District Assemblies (MMDAs) created under the Local Government Act 462 in 1993. This Municipality covers an area of 450km^2^. It is one of the twenty-six administrative districts in the Eastern region of Ghana. The Suhum Municipality consists of nine sub-municipals, namely; Akorabo, Akote, Ayekotse, Kukua, Nankese, Obretema, Sra, Suhum Central and Supresu.

It is located in the southern part of the Eastern region. Suhum, the municipal capital is about 60 kilometers north–west of Accra, the capital city of Ghana. It is bounded by East Akim to the north, Ayensuano to west and south, Akwapim North municipality to the east and New Juaben Municipality to North east. The Municipality has an approximated 113,885 population comprising of 55,628 and 58,257 males and females respectively [9].

Farming is the major occupation of occupants in the municipality followed by trading [10]. The sources of drinking water are pipe-borne in the township, boreholes in towns and villages as well as untreated water from the main river (river Densu). The river Densu is the largest water body within the Municipality and flows from the north to the south. The major rainy season is from May to July, and August to October is the minor rainy season whilst November to February is the dry season (harmattan).

The Municipality has a hospital, five private community clinics, five Government Health Centres and twenty-one Community-based Health Planning and Services (CHPS) compounds. The Municipal Health Directorate (MHD) to see to the health of the populace of the Municipality and its surrounding communities.

### Study population

A total of 840 persons were diagnosed with tuberculosis (TB) and registered in the Suhum Municipal TB registers from January 1, 2009 –December 31, 2018 and these constituted the study population for this study. Data of all TB patients entered into the Suhum Municipal TB registers from January 1, 2009 to December 31, 2018 were included in the study. All incomplete data in the municipal TB registers were excluded from the study.

### Study design

The study employed a descriptive cross-sectional design. We collected data from the Municipal TB register of all patients that attended the health facilities from December 31, 2018 to January 1, 2009.

### Data source and extraction

The Municipal TB register is the standard tool for capturing all data on TB patients across the country and it is usually kept by the municipal coordinator for the TB control programme. A self-designed data extraction form was developed using the 2015 Microsoft Excel spreadsheet.

The form was developed as line listing and had rows and columns with the columns having the indicators of interest. Each row captured individual information. Data were then extracted directly into the Microsoft Excel spreadsheet to capture all relevant variables. Data extractions were done by the principal investigator together with two other trained persons in the presence of the coordinator for the TB control programme. Variables such as sex, age, type of TB and patient category were extracted for analysis. Also, information on whether or not patients were screened for HIV and the test results of those screened with their respective dates of diagnosis were extracted.

### Data management and analysis

Data extracted into Microsoft Excel 2015 spreadsheet were cleaned to ensure completeness and exported into STATA version 16.0 for analysis. Data extracted were kept anonymous and only the investigators had access to the data. Results of the analysis were displayed in tables and graphs in relation with the study variables and these included socio-demographic information of TB patients, proportion of TB patients tested for HIV, proportion of HIV seropositive among those tested, proportion of TB and TB/HIV co-infected patients stratified by residence location, HIV status of TB patients stratified by age group, sex and sub-municipal. Continuous variables were summarized by median and interquartile range whiles categorical variables were summarized by frequencies and proportions. Line graphs were used to display the temporal trend of TB/HIV co-infection with proportions on the y-axis against the study periods on the x-axis.

### Data validity

The data were cleaned and checked for accuracy and completeness before exporting for analysis. The Municipal TB coordinator systematically checked and compared extracted data with the registers to ensure data quality and accuracy.

### Ethics statement

Ethical clearance was obtained from the University of Port Harcourt Research Ethics Committee **(UPH/CEREMAD/REC/MM70/001)** and the Ghana Health Service Ethics Review Committee (GHS-ERC) **(GHS-ERC 023/06/20)**. Also, a written permission to conduct the study was sought from the Municipal Health Management Team (MHMT) before commencement of the study. Nevertheless, the investigators had no competing interest. No consent was sought from TB patients whose records were reviewed because the study made use of secondary data. Additionally, data extracted for the purpose of this study was anonymously extracted as the extraction excluded identifiers of patients. Codes instead of names were used as identifiers for each record reviewed. Data extractions were however done in the presence of the Municipal TB control programme coordinator.

## Results

### Distribution of socio-demographic characteristics of study participants by year

Table 1 showed that overall, 840 TB patients were registered at Suhum Municipal from January 1, 2009-December 31, 2018. A greater proportion 146 (17.4%) were registered in 2012 with the least 41 (4.9%) in 2009. Of these 840, majority 527 (62.7%) and 541 (64.4%) were males and residence of urban areas respectively. Almost half of the participants 371 (44.2%) fell within the age group 30–49 years whereas 31 (3.7%) were <15 years. The Patients age ranged between 1–100 years with median age [interquartile range (IQR)] of 44(33–56) years.

**Table 1:** Demographic characteristics of TB patients in Suhum Municipality from 2009–2018.

Characteristics	2009	2010	2011	2012	2013	2014	2015	2016	2017	2018	Total
	[n = 41]	[n = 57]	[n = 52]	[n = 146]	[n = 97]	[n = 102]	[n = 94]	[n = 87]	[n = 79]	[n = 85]	[N = 840]
	n (%)	n (%)	n (%)	n (%)	n (%)	n (%)	n (%)	n (%)	n (%)	n (%)	N (%)
Median age yrs	36	40	40	44	41	43	46.5	47	45	45	44
(IQR)	(27–48)	(30–52)	(30–52)	(32–58)	(34–56)	(33–53)	(36–60)	(36–60)	(32–57)	(35–55)	(33–56)
**Sex**											
Male	24(58.5)	38(66.7)	31(59.6)	88(60.3)	62(63.9)	52(51.0)	58(61.7)	65(74.7)	56(70.9)	53(62.4)	527(62.7)
Female	17(41.5)	19(33.3)	21(40.4)	58(39.7)	35(36.1)	50(49.0)	36(38.3)	22(25.3)	23(29.1)	32(37.7)	313(37.3)
**Age group in years**											
<15	5(12.2)	3(5.3)	3(5.8)	3(1.4)	5(5.2)	3(2.9)	2(2.1)	1(1.2)	4(5.1)	3(3.5)	31(3.7)
15–29	7(17.1)	9(15.8)	9(17.3)	24(16.4)	13(13.4)	18(17.7)	10(10.6)	7(8.1)	10(12.7)	13(15.3)	120(14.3)
30–49	21(51.2)	29(50.9)	24(46.2)	58(39.7)	45(46.4)	46(45.1)	41(43.6)	42(48.3)	31(39.2)	34(40.0)	371(44.2)
50–69	5(12.2)	10(17.5)	14(26.9)	43(29.5)	21(21.7)	25(24.5)	26(27.7)	27(31.0)	25(31.7)	29(34.1)	225(26.8)
≥70	3(7.3)	6(10.5)	2(3.9)	19(13.0)	13(13.4)	10(9.8)	15(16.0)	10(11.5)	9(11.4)	6(7.1)	93(11.1)
**Area of residence**											
Rural	4(9.8)	13(22.8)	10(19.2)	75(51.4)	33(34.0)	35(34.3)	38(40.4)	25(28.7)	36(45.6)	30(35.3)	299(35.6)
Urban	37(90.2)	44(77.2)	42(80.8)	71(48.6)	64(66.0)	67(65.7)	56(59.6)	62(71.3)	43(54.4)	55(64.7)	541(64.4)

### HIV detection rate and prevalence of dual infection of TB and HIV from 2009–2018 by Sub-municipal

Table 2 showed that out of the 10 sub-municipals in Suhum Municipality, more than half 477 (56.8%) of the TB patients were registered at Suhum Central with Supresu registering the least 8 (1.0%).

**Table 2:** HIV test rate and prevalence of HIV among TB patients from 2009–2018 stratified by sub-municipal.

Sub-municipal	TB cases registered from 2009–2018 n (%)	HIV test done n (%)	HIV positive n (%)	95%CI
Akorabo	72(8.6%)	71(98.6)	8(11.1)	0.05–0.21
Akote	17(2.0)	16(94.1)	3(17.6)	0.04–0.43
Ayekotse	52(6.2)	50(98.2)	11(21.2)	0.11–0.35
Ayensuano	54(6.4)	50(92.3)	9(16.7)	0.08–0.30
Kukua	12(1.4)	11(91.7)	0(0.0)	0.00–0.26
Nankese	50(6.0)	47(94.0)	5(10.0)	0.33–0.22
Obretema	54(6.4)	49(90.7)	14(25.9)	0.15–0.40
Sra	44(5.2)	43(97.7)	8(18.2)	0.08–0.33
Suhum Central	477(56.8)	448(93.9)	97(20.3)	0.17–0.24
Supresu	8(1.0)	8(100)	1(12.5)	0.00–0.53
TOTAL	840(100)	793(94.4)	156(18.6)	0.16–0.21

Of the 840 TB patients registered over the study period, majority 793 (94.4%) were tested for HIV. Nevertheless, Supresu recorded a 100% HIV screening rate with the other 9 sub-municipals recording above 90% HIV test rate. Overall, 156 (18.6%) [(95%CI: 0.16–0.21)] of those tested for HIV were positive.

### HIV status of TB patients in Suhum Municipal stratified by age group in years

**Fig 2:**
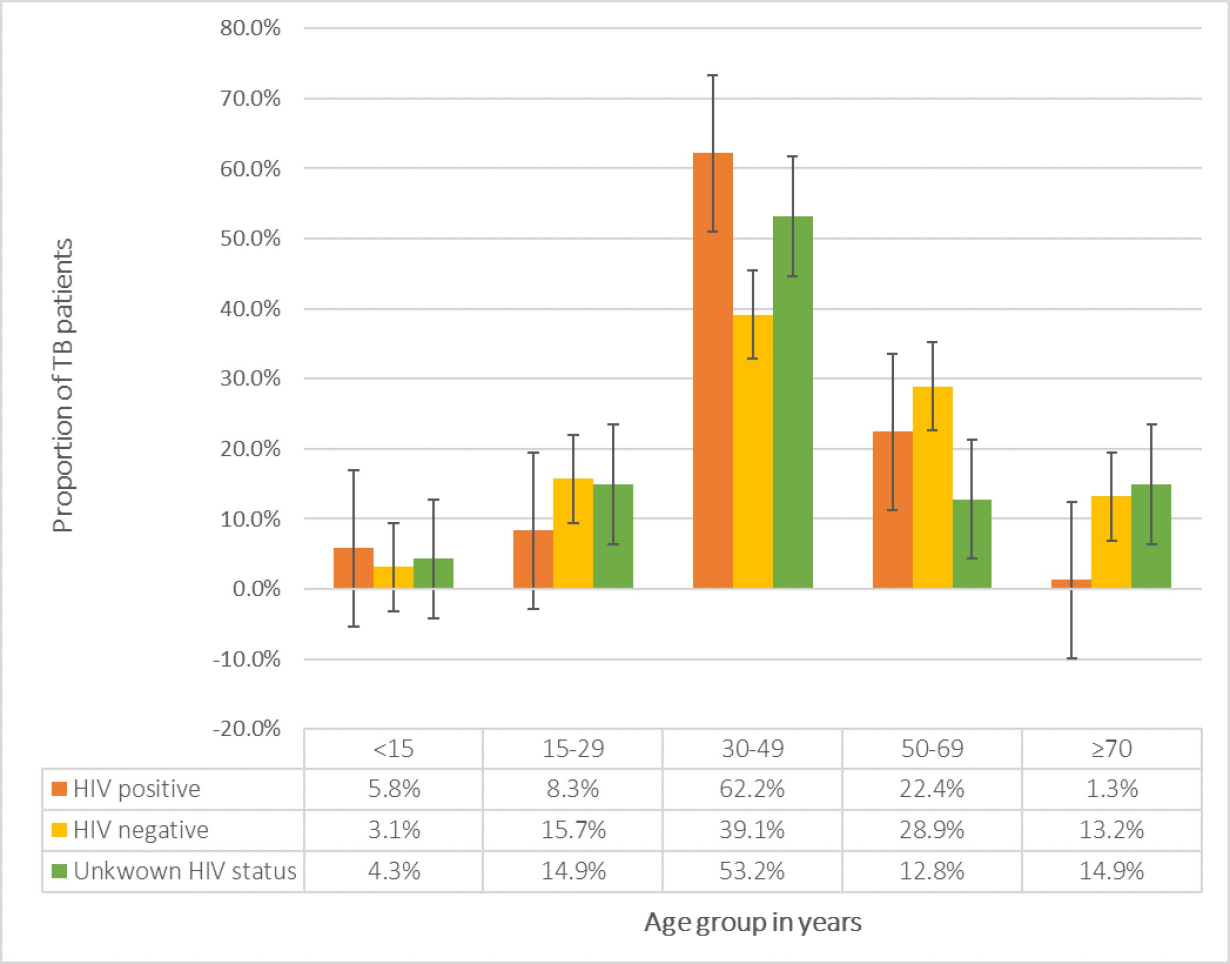
HIV status of TB patients in Suhum Municipal stratified by age group.

Fig 2 indicated that proportion of TB/HIV co-morbidity was highest 97 (62.2%) among TB patients within the age bracket 30–49 years with the least 2 (1.3%) among those ≥70 years.

### HIV status of TB patients stratified by sex in Suhum Municipal from 2009–2018

**Fig 3:**
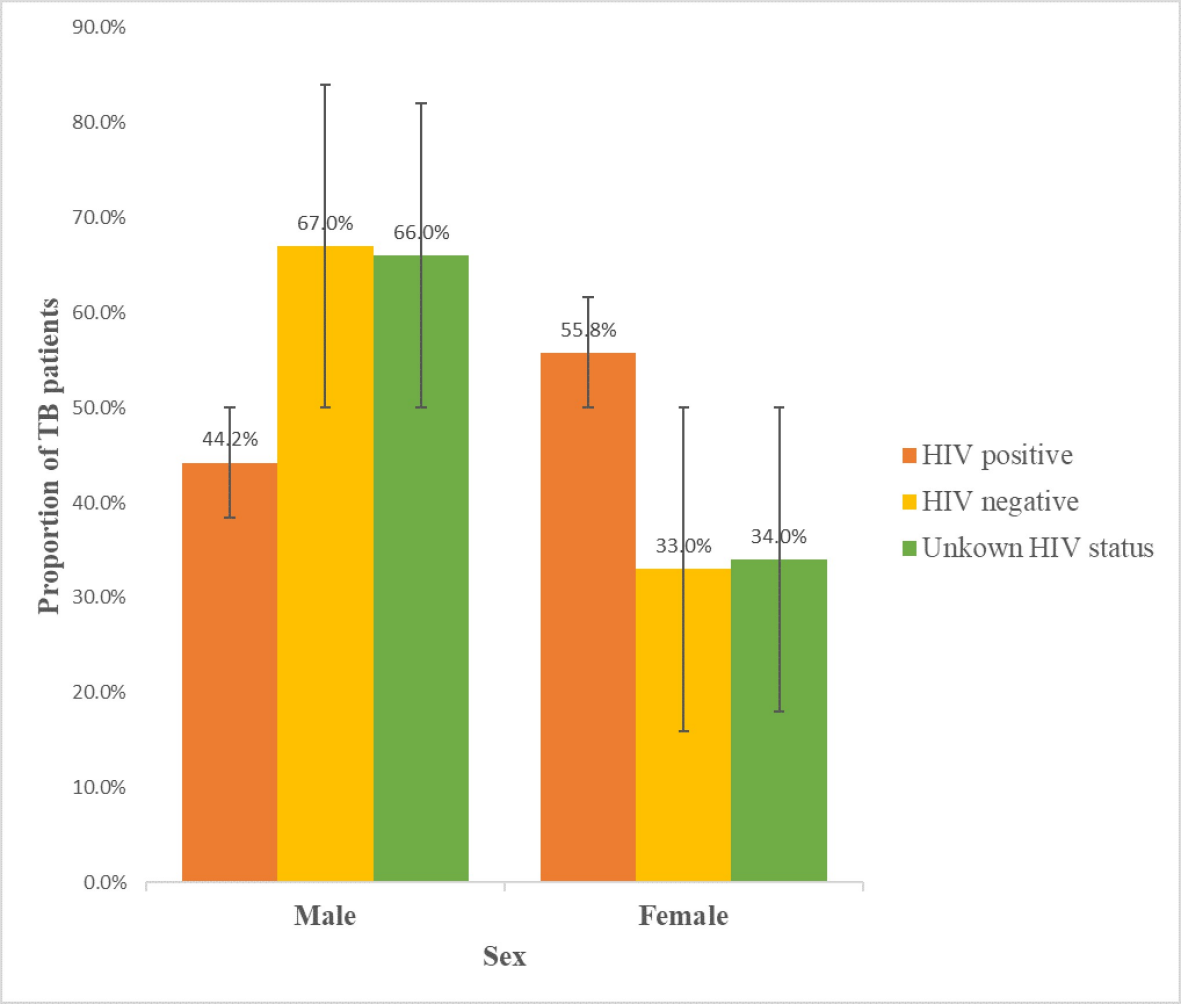
HIV status of TB cases by sex in Suhum Municipal.

Fig 3 Showed that overall, the prevalence of TB/HIV co-infection was highest 87 (55.8%) for females during the study period. Also, 16 (34.0%) of female TB patients were not screened for HIV.

### Prevalence of TB/HIV co-infection in Suhum Municipal by age group in years

**Fig 4:**
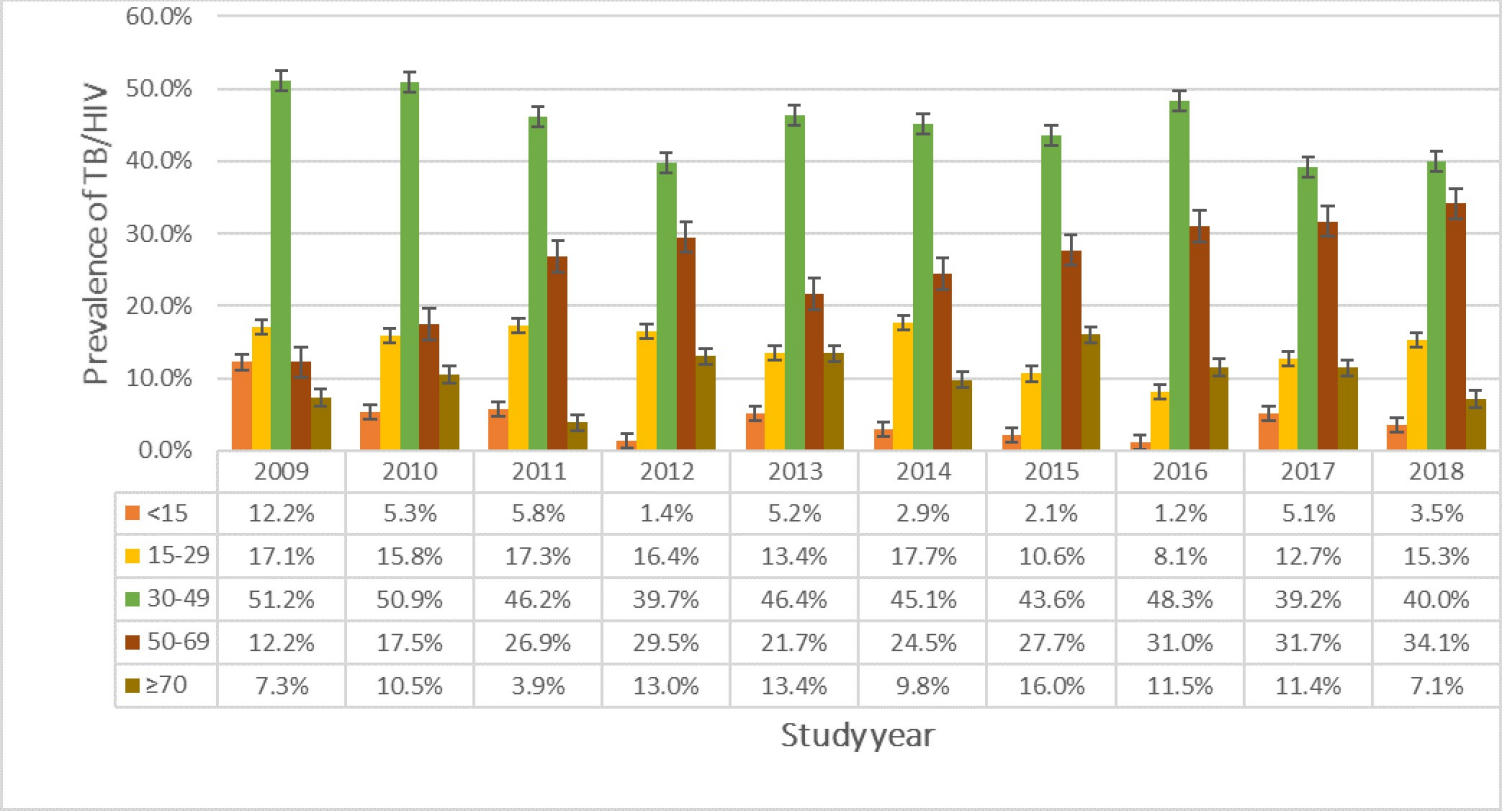
Annual prevalence of TB/HIV co-infection in Suhum Municipal stratified by age group.

As detailed in Fig 4 the prevalence of TB/HIV co-infection was fairly stable over the 10-year period for the age groups 15–29 years, 30–49 years and ≥ 70 years. Nonetheless, the age groups <15 years and 50–69 years witnessed a reduction and an increment respectively in the prevalence of the dual infection over the study period.

### Temporal trend of TB/HIV co-infection in Suhum Municipal from 2009–2018

**Fig 5:**
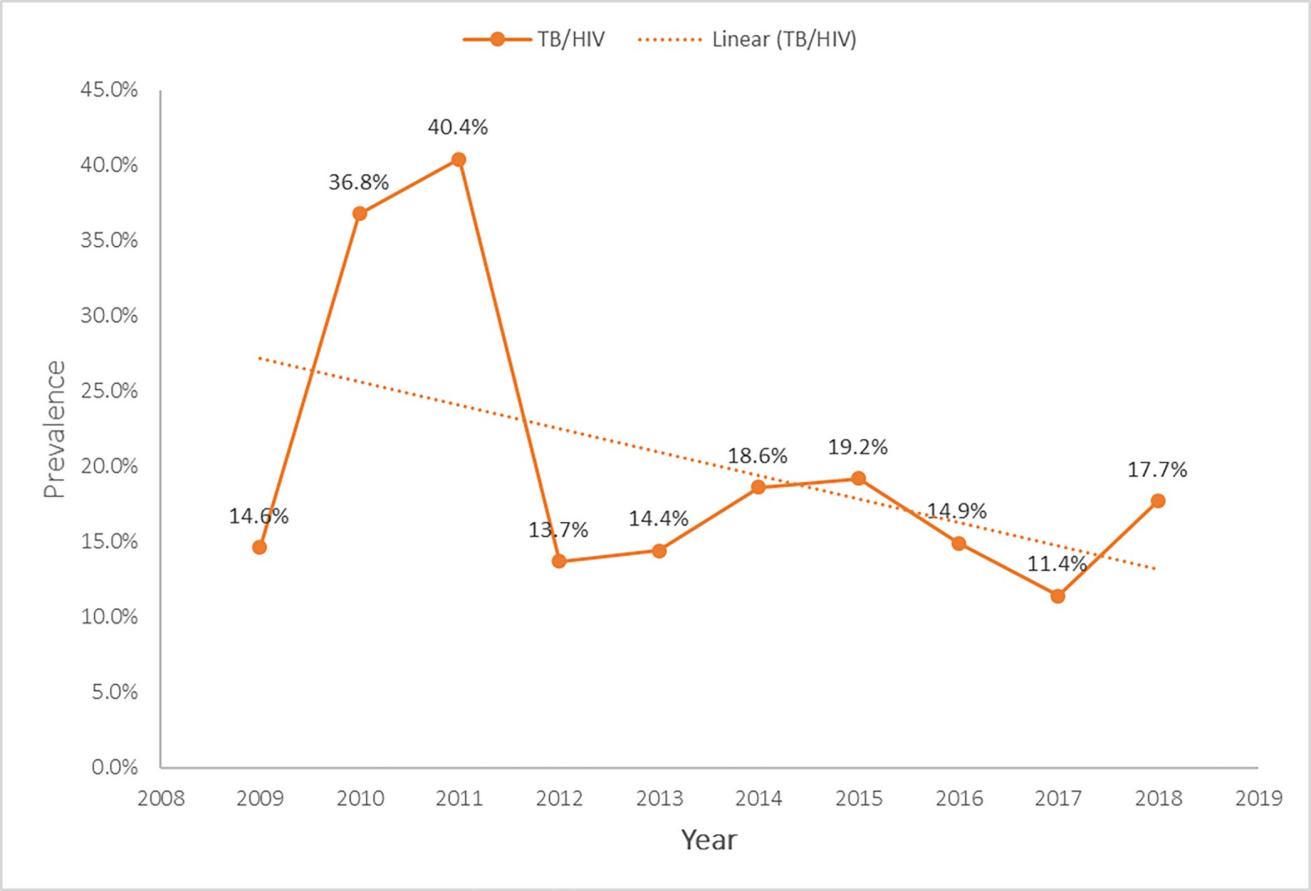
Trend of TB/HIV co-infection in Suhum Municipal from 2009–2018.

According to Fig 5, there was a sharp increase in the proportion of the co-morbidity from 6 (14.6%) in 2009 to 21 (36.8%) in 2010, a sharp decrease was however recorded from 21 (40.4%) in 2011 to 21 (13.7%) in 2012. The highest proportion of 21 (40.4%) observed in 2011 with the least 9 (11.4%) in 2017. There was however, an overall downward trend of the co-morbidity during the study period.

## Discussion

### HIV test rate among TB patients

This study established that a high proportion (94.4%) of TB patients had HIV screening done in keeping with the World Health Organization (WHO) protocol. It is an indication that quality processes are employed in TB clinics in this Municipality. This report from Suhum municipal was a major improvement over the global and WHO African Region’s documented HIV test results of 69% and 86% respectively in 2019 [2] and that of the national estimate of 93% in 2018 [11].

In a study in another part of Ghana, the Volta region found that 92.2% of the 1,772 TB cases were screened for HIV between 2012 and 2015 [5]. Several reports from other parts of Ghana and other countries under the WHO African region showed very high HIV screening rates among persons living with TB as Imo State Nigeria, reported 89%, Malawi 95%, South Africa 92.9%, Addis Ababa, Ethiopia 92.0% [12–15]. The high-test rates are a strong pointer to a good commitment from health professionals and functional control measures [16].

### Prevalence of HIV among TB patients

Out of the 793 TB patients that were tested for HIV, an overall 18.6% were co-infected with HIV. Greater proportions of the co-morbidity were however, found among TB patients aged 30–49 years and female TB patients (62.2% and 55.8% respectively). A possible contributory factor to this observation could be due to the fact that the age bracket 30–49 years constitutes the active age as well as sexually active age. The biological composition of the female reproductive system makes them more susceptible to the risk of acquiring HIV infection [17]. Also, women tend to have a better health seeking behaviour compared with men therefore are usually able to approach the healthcare system easily and access screening earlier than men [18, 19].

The current finding agrees with the 2018 national co-infection prevalence of 19.0% and that of the Volta region of Ghana where an overall prevalence of HIV co-infection among TB patients were 18.2% and 19.1% in 2017 and 2019 respectively [5, 8]. Nonetheless, the prevalence of TB/HIV co-infection observed in Suhum Municipal was much lower than the Eastern regional prevalence of 33.4% in 2014 [20] and the WHO African Region estimated prevalence of 29% in 2018 [11]. Nonetheless, the observation made in this recent study is much higher than the global estimated prevalence of 8.2% established in 2019 [2]. This indicates that HIV is also prevalent in the Volta region of Ghana but lower than the Eastern part of the country [21].

Other parts of Africa showed much variation. A more recent study in Kano State, Nigeria found the prevalence of the co-infection to be lower (11.2%) than the observation made in this study. Also, a 21.6% TB/HIV co-infection rate was identified in a study in Lagos, Nigeria [22]. Contrary to findings of this study, a study in Abeokuta, Ogun State, Nigeria recorded no TB/HIV co-infection among their study participants [23]. The Ogun state study tallies with a rural study which reported less than 2.0% prevalence of HIV among persons with TB in south-south, Nigeria [24]. In describing further variations, a 2018 report from reference Genexpert sites in River’s state, Nigeria differed from the current finding. Their report noted 32.7% prevalence of HIV among TB patients [25]. Other reports were 42.7% and 56% co-infection rates from Kenya and Malawi respectively [15, 26].

### Temporal trend of TB/HIV co-infections

This study observed a relatively stable prevalence trend hovering between 14.6% and 19.2%. During the initial years under the study, there was spike over two consecutive years (2010 and 2011) which corresponded with strengthening of the TB/HIV collaborative activities and thereafter a dip in 2012 followed by a stable trend until 2018. Nevertheless, this study pointed to an overall decrease in the temporal trend of the dual infection over the study period. Although, a number of factors could explain the decreasing trend of the co-morbidity seen, however, it could be due to the decentralized nature of TB care in Ghana coupled with better adherence to TB and HIV care.

Overall, the downward trend of the co-infection observed at the Suhum Municipal is consistent with other evidences from Ethiopia and Kenya [27–29] but varied markedly from Northwest Ethiopia [30] and Urumqi, Xinjiang Province, China [31] where fluctuating trends of the co-infection were observed. In contrary, a stable trend of the co-infection was noted in similar report from Ghana [32].

Different reports on the trend of the co-infection emerged from a study in Sierra Leone where the incidence of the dual infection increased steadily [33]. This observation is suggestive that TB and HIV activities are either not collaborated or intensified in this part of Sierra Leone compared to Suhum Municipality of Ghana.

Nevertheless, the study provided useful information to be used for TB and HIV programme planning and implementation.

### Conclusion and recommendation

In conclusion, the study revealed a good HIV test rate among those with TB in Suhum Municipal in Ghana, this is very commendable and must therefore be improved on and efforts sustained. This indicates that TB patients receive quality care in the Eastern region of Ghana, more particularly in the Suhum Municipality. More so, it implies there is a better commitment from the Municipal Health Directorate (MHD) and their health professionals in terms of communication and social mobilization.

The proportion of TB/HIV co-infection in this study was relatively high compared with similar studies in the country. The co-morbidity was however, quite prevalent among the female sex and 30–49 years age bracket. Additionally, the results further points to a decrease trend of the co-infection over the study period.

There is the need also for sex and age targeted interventions lead by the Ghana Health Service and the Health Ministry, such as creating of awareness for females and persons that fall within the working class and sexually active age bracket in the country on the effect of these two infections on productivity and national development, especially across the Eastern region. Continuous efforts should therefore be geared towards the decentralized nature of the TB control programme in the country by the Health Ministry and the Ghana Health Service so as to maintain a decreasing trend of the co-infection in the years ahead.

## Supporting information

S1 Data This is the minimal anonymized data set used for the study and also necessary to replicate the study. (XLSX)
